# Promoting therapeutic angiogenesis of focal cerebral ischemia using thrombospondin-4 (TSP4) gene-modified bone marrow stromal cells (BMSCs) in a rat model

**DOI:** 10.1186/s12967-019-1845-z

**Published:** 2019-04-04

**Authors:** Qian Zhang, Meiling Zhou, Xiangfeng Wu, Zhu Li, Bing Liu, Wenbin Gao, Jin Yue, Tao Liu

**Affiliations:** 1 Department of Biotherapy and Oncology, Shenzhen Luohu People’s Hospital, Shenzhen, 518001 Guangdong People’s Republic of China; 2 Public Service Platform for Cell Quality Testing and Evaluation of Shenzhen, Shenzhen, 518001 Guangdong People’s Republic of China; 3 Mudanjiang Medical University, Mudanjiang, 157011 Heilongjiang People’s Republic of China; 4 The 230th Hospital of the Chinese PLA, Dandong, Liaoning People’s Republic of China

**Keywords:** Thrombospondin-4, Bone marrow stromal cells, Angiogenesis, Cerebral ischemia

## Abstract

**Background:**

A stroke caused by angiostenosis always has a poor prognosis. Bone marrow stromal cells (BMSC) are widely applied in vascular regeneration. Recently, thrombospondin-4 (TSP4) was reported to promote the regeneration of blood vessels and enhance the function of endothelial cells in angiogenesis. In this work, we observed the therapeutic effect of TSP4-overexpressing BMSCs on angiogenesis post-stroke.

**Methods:**

We subcloned the *tsp4* gene into a lentivirus expression vector system and harvested the *tsp4* lentivirus using 293FT cells. Primary BMSCs were then successfully infected by the *tsp4* virus, and overexpression of GFP-fused TSP4 was confirmed by both western blot and immunofluorescence. In vitro, TSP4-overexpressing BMSCs and wild-type BMSCs were co-cultured with human umbilical vein endothelial cells (HUVECs). The expression level of TSP4, vascular endothelial growth factor (VEGF) and transforming growth factor-β (TGF-β) in the supernatant were detected by enzyme-linked immunosorbent assay (ELISA). Wound healing, tube formation and an arterial ring test were performed to estimate the ability of TSP4-overexpressing BMSCs to promote the angiogenesis of endothelial cells. Using a rat permanent middle cerebral artery occlusion (MCAO) model, the effect of TSP4-overexpressing BMSCs on the regeneration of blood vessels was systematically tested by the neurological function score, immunohistochemistry and immunofluorescence staining assays.

**Results:**

Our results demonstrated that TSP4-overexpressing BMSCs largely increased the expression of VEGF, angiopoietin-1 (Ang-1), matrix metalloprotein 9 (MMP9), matrix metalloprotein 2 (MMP2) and p-Cdc42/Rac1 in endothelial cells. TSP4-BMSC treatment notably up-regulated the TGF-β/Smad2/3 signalling pathway in HUVECs. In vivo, the TSP4-BMSC infusion improved the neurological function score of MCAO rats and expanded the expression of the von Willebrand factor (vWF), Ang-1, MMP2 and MMP9 proteins in cerebral ischemic penumbra.

**Conclusions:**

Our data illustrate that TSP4-BMSCs can promote the proliferation and migration of endothelial cells and tube formation. We found that TSP4-BMSC infusion can promote the recovery of neural function post-stroke. The *tsp4* gene-modified BMSCs provides a better therapeutic effect than that of wild-type BMSCs.

**Electronic supplementary material:**

The online version of this article (10.1186/s12967-019-1845-z) contains supplementary material, which is available to authorized users.

## Background

Stroke is a common disease in modern society, especially in the middle-aged and elderly populations, and is one of the three most fatal diseases in humans along with heart disease and malignant tumours. In recent years, the incidence rate of stroke has been increasing, with a tendency of affecting youth [1]. Ischemic stroke is a common type of stroke that is characterized by high disability and high mortality. Ischemic stroke accounts for approximately 70% of strokes in China [2, 3], while the proportion in Europe and the United States is as high as 85%. Statistical results show that three-quarters of the ischemic stroke patients are lost from the labour force, and two-fifths of the patients have a severe disability [4, 5]. Therefore, it is of great research significance and social value to prevent and treat ischemic stroke. Ischemic stroke prevention and treatment reduce the incidence of ischemic stroke and promote the recovery of nerve function in stroke patients.

The main pathological mechanism of ischemic stroke is the stenosis and occlusion of an intracranial artery, resulting in ischemic and hypoxic necrosis of neurons in the corresponding brain tissues. Therefore, how to improve blood circulation after cerebral ischemia is an active research area in the treatment of ischemic stroke. With the advancement of stem cell therapy, a variety of stem cell lines have been used in recent studies of cerebral ischemia [6, 7]. Bone marrow stromal cells (BMSCs) are a group of cells with the characteristics of self-renewal, high proliferation ability and multilineage differentiation. BMSCs transplantation, especially through intra-arterial delivery, can effectively improve neurological function intra-arterial, promote synaptogenesis, endogenous cell proliferation, axon regeneration, alleviate neuron/axon injury, promote the proliferation of oligodendrocyte progenitor cells (opcs) and the formation of myelin sheath, and alleviate white matter injury in rats with focal cerebral ischemia [8, 9]. At present, phase I and II clinical trials of autologous BMSC transplantation for ischemic stroke approved by the FDA in the United States have been completed and demonstrate the feasibility of autologous BMSC transplantation for cerebral ischemia via an intravenous route [10]. Although BMSCs are widely reported to functionally elevate blood circulation in post-stoke treatment, the clinical application of BMSCs remains restricted due to insufficient angiogenesis promoting factor secretion from the limited number of BMSCs [7, 10].

Thrombospondin-4 (TSP4) is a member of the thrombospondin (TSP) family, which is a multifunctional matrix glycoprotein with a molecular weight of 550 kD. TSP4 is secreted mainly from the myocardium and smooth muscle and can be combined with many types of glycoproteins. Matrix proteins can be bound to regulate cell–cell–matrix interactions and participate in biological processes, such as platelet adhesion and aggregation, thrombosis, smooth muscle proliferation and migration [11–13]. In vivo and in vitro experiments have shown that TSP4 can promote neovascularization and enhance the angiogenesis-promoting function of endothelial cells [14], and its expression is closely related to transforming growth factor-β (TGF-β) [15]. Therefore, in this study, we designed experiments to evaluate whether the overexpression of TSP4 in BMSCs can promote angiogenesis and further improve the clinical efficacy of BMSC transplantation in the treatment of ischemic stroke.

## Materials and methods

### BMSC culture and identification

Male Sprague–Dawley (SD) rats (Guangdong Medical Laboratory Animal Center, Foshan, Guangdong, China) weighing 50–60 g were sacrificed by cervical dislocation. The tibias and femurs were removed and washed with phosphate buffer saline (PBS) 3 times, and the bone marrow was extracted by syringe needles. The freshly isolated cells were cultured with Dulbecco’s modified Eagle’s medium (DMEM)/F12 cell culture medium (Life Technologies, USA) supplemented with 10% foetal bovine serum (FBS) and 100 U/ml gentamycin. Twenty-four hours later, the culture medium was replaced.

At passage 3, digested BMSCs were incubated with fluorescence-conjugated antibodies, including CD90-PerCP, CD34-PE, CD45-Alexa (Santa Cruz, USA) and PBS (negative control), in a black chamber at 4 °C for 30 min. Then, the cells were washed with PBS and fixed in 4% paraformaldehyde, and flow cytometry using the Cellquest system (Becton, Dickinson and Company, USA) was performed to analyse purity. Flow cytometry analysis is described in detail in Additional file 1.

### Construction of the *PLV*-*Easy*-*gfp*-*tsp4* plasmid

The PCR conditions were 2 min of pre-denaturation at 94 °C; 30 reaction cycles of 10 s denaturation at 94 °C, 30 s annealing at 57 °C and 3 min extension at 72 °C; followed by 5 min of final extension at 72 °C. The PCR primer sequences were as follows: forward: 5′-CGGGATCCATGCCGGCCCCAC-3′ reverse: 5′-CCGCTCGAGATTATCCAAGCGGTC-3′. The plasmid was digested with the *Bam*HI and *Xho*I enzymes to confirm that the plasmid was the correct size. Sequencing analysis of the plasmid was completed by Bioengineering (Shanghai) Co., Ltd.

### *PLV*-*Easy*-*gfp*-*tsp4* lentivirus preparation and BMSC infection

The 293FT cells were co-transfected with the recombinant lentiviral vector and two auxiliary packaging plasmids (psPAX2 and pMD.2G), followed by cell culture for 48 h and 72 h. Then, the supernatant was collected from the cells and filtered through a 0.45 μm membrane. Finally, the recombinant lentiviral vector (*PLV*-*Easy*-*gfp*-*tsp4*) containing *tsp4* and the green fluorescent protein reporter gene (*gfp*) was obtained.

BMSCs were gently washed twice with PBS before infection, and the harvested lentivirus, 8 mg/ml polybrene and DMEM/F-12 were added in an appropriate ratio. The medium (DMEM/F-12 + 10% FBS + 1‰ GM) was replaced after 6 h. The infection efficiency was observed on the third day. BMSCs infected by lentivirus were split (0.25% trypsin/1 mM EDTA) and further enriched by passage cultures. Flow cytometry (Becton–Dickinson, USA) was performed to identify CD44, CD90, CD45 and CD34 surface markers on the cultured cells. Flow cytometry analysis is described in detail in Additional file 1.

### Cellular immunofluorescence staining

BMSCs and TSP4-BMSCs were plated into 24-well plates (4 × 10^5^ cells per well) and were cultured in an incubator overnight. The cells were fixed with 4% paraformaldehyde for 20 min and washed twice with PBS. The cells were stained with Phalloidin (1:500) for 60 min and DAPI (1:1000) for 10 min in the dark. Then, the cells were photographed using a fluorescence inverted microscope (Axio Observer 3, Carl Zeiss AG).

### Enzyme-linked immunosorbent assay

Human umbilical vein endothelial cells (HUVECs, ATCC^®^ CRL-1730) were co-incubated with BMSCs and TSP4-BMSCs, and the medium was collected after 48 h (including HUVECs only). According to the manufacturer’s instructions (ELISA KIT Manual of Beijing Sizheng Bai Biotechnology Co., Ltd.), various solutions were prepared. Three groups of samples (HUVEC, BMSC + HUVEC, TSP4-BMSC + HUVEC) and different concentration standards were added to the corresponding wells. VEGF, TGF-β and TSP4 biotinylated antibodies and the corresponding enzymes were added for 60 and 90 min, respectively, and the reaction wells were washed 4 times. Developers were added for 10–20 min at 37 °C in the dark. After stopping the reaction, the optical density at 450 nm was detected using an enzyme labelling apparatus.

### Arterial ring experiment

The thoracic cavity of adult rats was exposed using surgical tools. The thoracic aorta was dissected and cut into slices of approximately 1 mm and embedded in 48-well plates coated with Matrigel. Three groups cell culture supernatant was added in each well, and duplicate wells were set for each group. After labelling, the cells were cultured in a cell culture incubator, and an image was taken using an inverted phase contrast microscope (Axio Observer 3, Carl Zeiss AG) at 72 h.

### Tube formation assay

BD Matrigel matrix was plated in a pre-cooled 48-well chamber, and the chamber was transferred for solidification at 37 °C, 5% CO_2_ for 1 h. HUVECs were trypsinized and seeded (5 × 10^4^ cells per well) with 10 µg/ml recombinant FN (Sigma, USA). The chambers were then incubated at 37 °C for 2 days. The supernatant of TSP4-BMSCs and BMSCs and control medium were added to HUVECs for 48 h. Tube formation was photographed using a phase-contrast microscope, and the number of branch points were counted to verify the extent of angiogenesis in each group.

### The establishment of a middle cerebral artery occlusion model and experimental group

The present investigation conforms to the National Institutes of Health Guide for the Care and Use of Laboratory Animals (NIH Publications No. 80-23), revised in 1996. Male SD rats (n = 60) weighing 240–260 g and supplied by the Guangdong Medical Laboratory Animal Centre (Guangdong, China) were anesthetized with 10% (w/v) chloral hydrate (3.0 ml/kg, i.p.), and a permanent middle cerebral artery occlusion (MCAO) model was established according to the method described by Longa and colleagues [16].Video clip of the disease model and disease amilioration rat model has been enclosed in the Additional file 2. Additional file 3 is the video clip of control animal. Briefly, the middle cerebral artery of SD rats was obstructed with a 4–0 surgical nylon suture (length of 20–22 mm, determined by body weight) coated with polylysine, which was inserted into the internal carotid artery from the external carotid artery. A 5-point scale neurological deficit score as reported by Yilmaz and colleagues [17] was used to evaluate the MCAO model. Only animals with a score of 2 (circling to the right) were selected for group division.

A total of 60 MCAO rats were included in this study, and the rats were randomly divided into: 1) MCAO group (n = 20), 2) BMSC group (n = 20), and 3) TSP4-BMSC group (n = 20). One millilitre of PBS, BMSC or TSP4-BMSC suspension solution (2 × 10^6^ cells/ml) was injected into MCAO rats via the caudal vein at 3 h after model establishment. A sham-operated group (n = 20) was also established to exclude the influence of the operation process on the therapeutic effect.

### Immunohistochemistry and immunofluorescence staining

On day 28 after the operation, the rats in the MCAO, BMSC and TSP4-BMSC groups were sacrificed for histological studies. Rat brains (n = 6) were fixed with 4% paraformaldehyde, embedded in paraffin and cut into a series of 5-μm thick sections. For morphological analysis of vessels, samples were rinsed with PBS (Sigma, USA) containing 0.01% Tween-20 and immersed in 3% H_2_O_2_/methanol for 15 min to inhibit endogenous peroxidase activity. Subsequently, brain sections were incubated with 10% normal goat serum for 20 min at room temperature and then incubated with primary antibodies against vWF (1:200) and Ang-1 (1:500) at 4 °C overnight. Following an incubation with secondary antibody, sections were developed with a DAB kit and then stained with haematoxylin as a counterstain. After the transparent treatment, an image was obtained using an inverted phase contrast microscope (Axio Observer 3, Carl Zeiss AG). von Willebrand factor (vWF) and Angiopoietin-1 (Ang-1) expression were quantified using Image-Pro Plus software in the ischemic boundary zone (IBZ). To identify the expression of matrix metalloprotein 2 (MMP2) and matrix metalloprotein 9 (MMP9) in the IBZ, the slices were incubated with rabbit primary antibodies against MMP2 (1:50) and MMP9 (1:200) at 4 °C overnight.

### Statistical analysis

Statistical calculations were performed with Statistical Product and Service Solutions (SPSS) (version 17.0, Chicago, IL, USA) by one-way analysis of variance followed by a least significant difference test for multiple comparisons. Data were expressed as the mean ± standard deviation (SD). Differences were considered to be statistically significant at *p *< 0.05.

## Results

### BMSC morphology and characterization

Thy-1 (CD90) is a BMSC marker [18]; CD45 and CD34 are hematopoietic cell markers [19]. Cultured cells at passage 3 were used to determine the purity of BMSCs by flow cytometry. Additional file 1: Figure S1a shows that 2.74% and 3.55% of the cultured cells expressed CD45 and CD34, respectively, whereas the CD90 positive rate was as high as 99.76%. This result showed that high-purity BMSCs could be obtained in our study.

### Construction of the *PLV*-*Easy*-*gfp*-*tsp4* plasmid and expression of the TSP4 target protein in BMSCs

**Fig. 1:**
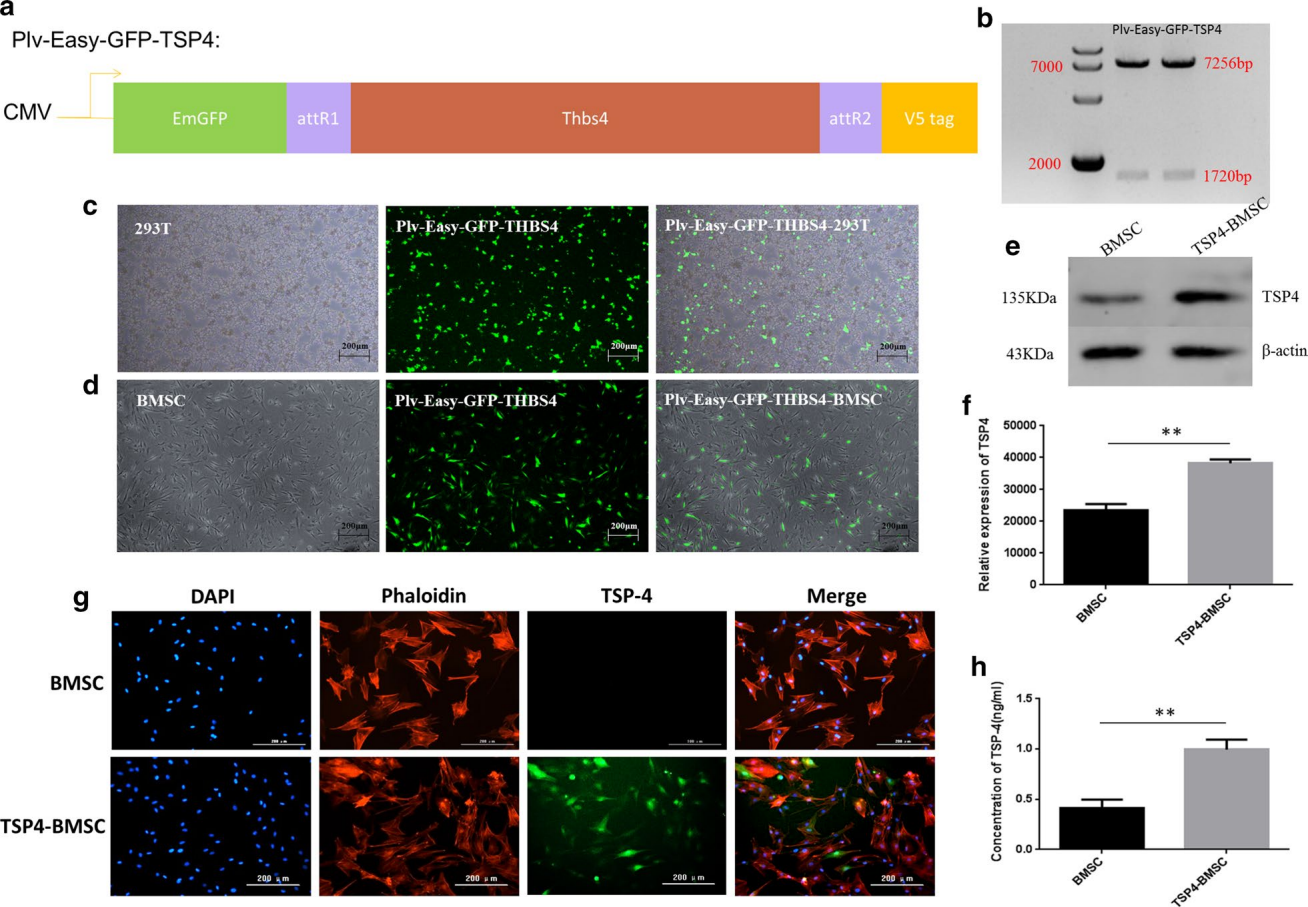
Construction of the PLV-Easy-GFP-TSP4 plasmid and expression of the TSP4 target protein. **a** Schematic illustration of the structure of PLV-Easy-GFP-TSP4. **b** *Sal*I and *Hin*dIII double digestion of two recombinant plasmids into 7256 and 1720 bp fragments. The positions of the 7000 and 2000 bp size markers are indicated to the left of the panel. **c** The plasmids psPAX2, pMD.2G and PLV-Easy-GFP-TSP4 were transfected into 293T cells (green) in the appropriate proportions. The positive rate of transfection was 94.73% ± 0.0126. **d** The PLV-Easy-GFP-TSP4 lentivirus infected BMSCs (green). The positive rate of infection was 76.95% ± 0.0278. **e** Fluorescent staining of PLV-Easy-GFP-TSP4-BMSCs and BMSCs (nucleus in blue, cytoskeleton in red, and TSP4 protein in green). **f** The TSP4 content in the supernatant of BMSCs was detected by ELISA. Data are expressed as the mean ± SD. **p < 0.01. **g** Western blot to determine the expression of TSP4 secreted by BMSCs in the two groups (BMSCs and TSP4-BMSCs). **h** Western blot results of TSP4 and quantitative analysis. Data are expressed as the mean ± SD. **p < 0.01

Figure 1a demonstrates a map of the recombinant *PLV*-*Easy*-*gfp*-*tsp4* lentiviral plasmid. Distinct bands were observed at 7256 bp and 1720 bp after agarose gel electrophoresis of PCR products from the transfected recombinant *PLV*-*Easy*-*gfp*-*tsp4* lentiviral plasmid-positive clones. The band size agreed with the expected results. The sequencing results agreed with the given *tsp4* gene sequence, suggesting that the recombinant *PLV*-*Easy*-*gfp*-*tsp4* lentiviral plasmid was successfully constructed (Fig. 1b). The transfection efficiency of the *gfp*-positive cells was detected 48 h after the 293FT cells were transfected with the recombinant *PLV*-*Easy*-*gfp*-*tsp4* plasmid. The percentage of *gfp*-positive cells was 94.73% ± 0.0126, indicating that the transfection was successful (Fig. 1c). The infection efficiency of the *gfp*-positive cells was detected at 72 h after the BMSCs were infected with the recombinant lentivirus vector. The percentage of *gfp*-positive cells was 76.95% ± 0.0278, indicating that the infection was successful (Fig. 1d). The western blot results showed that the expression of TSP4 in TSP4-BMSCs was significantly higher than that of normal BMSCs (**p < 0.01) (Fig. 1e, f). Fluorescent staining indicated that TSP4 protein was expressed in TSP4-BMSCs and was mainly present in the cytoplasm (Fig. 1g). The ELISA results showed that the TSP4 protein could be secreted extracellularly by TSP4-BMSCs, and the secretion of TSP4 in TSP4-BMSCs was significantly more than that of normal BMSCs (**p < 0.01) (Fig. 1h). The above results indicated that *tsp4* gene fragments were inserted into BMSCs, and the TSP4 protein could be overexpressed both inside and outside of the cells.

### TSP4-BMSCs promoted the proliferation and migration of HUVECs

**Fig. 2:**
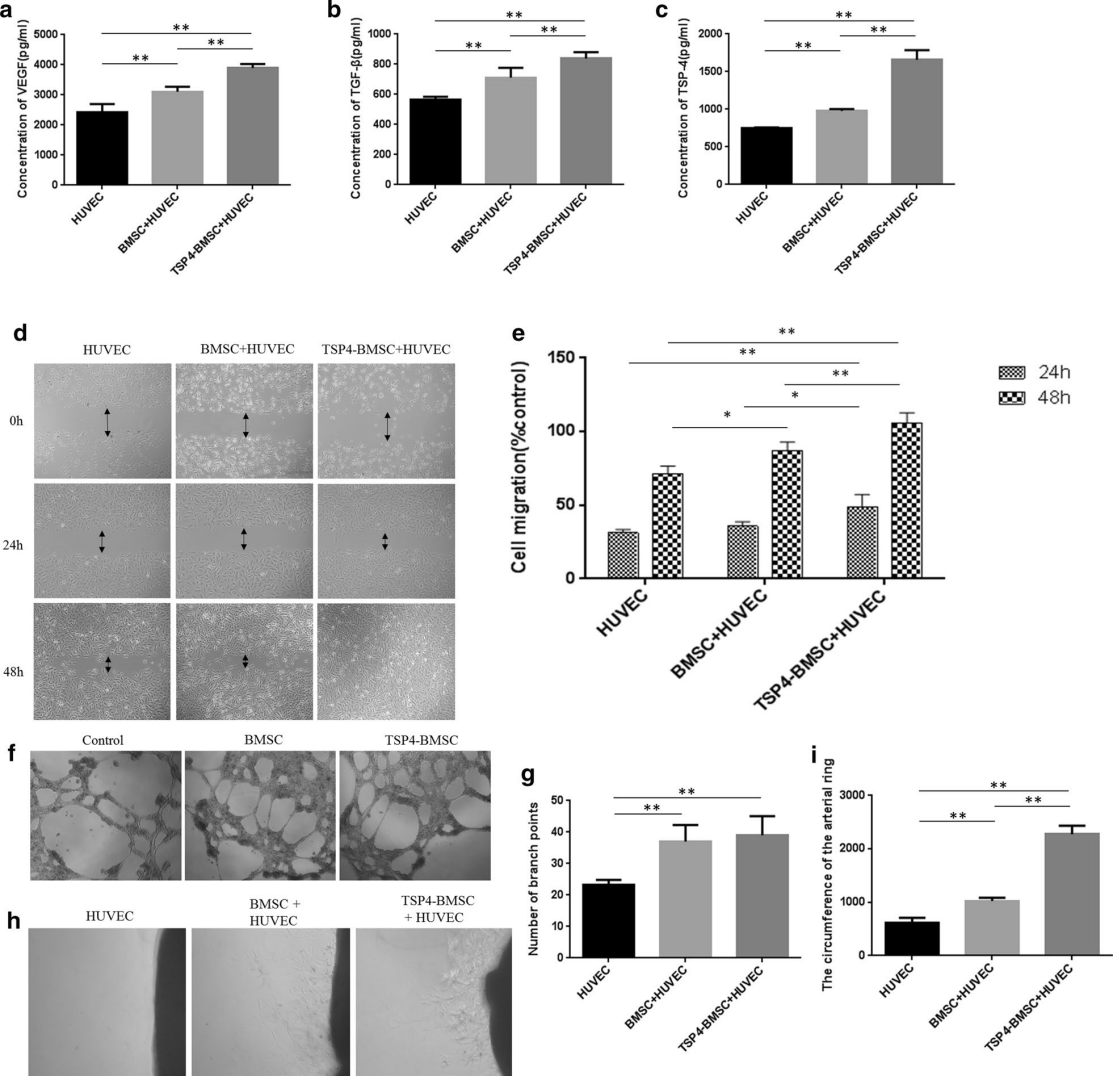
Effect of TSP4-BMSCs on HUVEC angiogenesis. **a**–**c** VEGF, TGFβ and TSP4 level in the supernatant of HUVECs incubated with BMSCs was detected by ELISA. Data are expressed as the mean ± SD. **p < 0.01. **d**, **e** Effect of TSP4-BMSCs on the proliferation and migration of HUVECs at 24 h and 48 h by wound healing test. Data are expressed as the mean ± SD. **p < 0.01, *p < 0.05. **f**, **g** Effect of TSP4-BMSCs on HUVEC tube formation at 48 h. Data are expressed as the mean ± SD. **p < 0.01. **h**, **i** The effect of TSP4-BMSCs on angiogenesis was examined by arterial ring experiment. Data are expressed as the mean ± SD. **p < 0.01

To clarify whether TSP4-BMSCs promote the proliferation and migration of endothelial cells, we performed a wound healing assay by incubating HUVECs with different conditioned media (CM). As shown in Fig. 2d, e, the wound healing assay showed that HUVECs incubated in the CM of the TSP4-BMSC group exhibited significantly higher cell migration than the cells incubated with the CM of control and BMSCs at 24 h and 48 h (**p < 0.01, *p < 0.05). This result demonstrated that treatment with TSP4-BMSCs improved the proliferation and migration of endothelial cells in vitro.

### TSP4-BMSCs contributed to angiogenesis and capillary formation of HUVECs

To investigate the potential roles of TSP4-BMSCs in angiogenesis in vitro, we performed arterial ring and tube formation assays by incubating HUVECs with different CM. HUVECs incubated with the CM of the TSP4-BMSC group showed significantly more endothelial tube formation and neovascularization than those incubated with the CM of the control and BMSC groups at 48 h (**p < 0.01, *p < 0.05) (Fig. 2f–i). These data indicated that TSP4-BMSCs can directly advance the angiogenic capability of endothelial cells.

### TSP4-BMSCs enhanced the expression of angiogenic factors in HUVECs

**Fig. 3:**
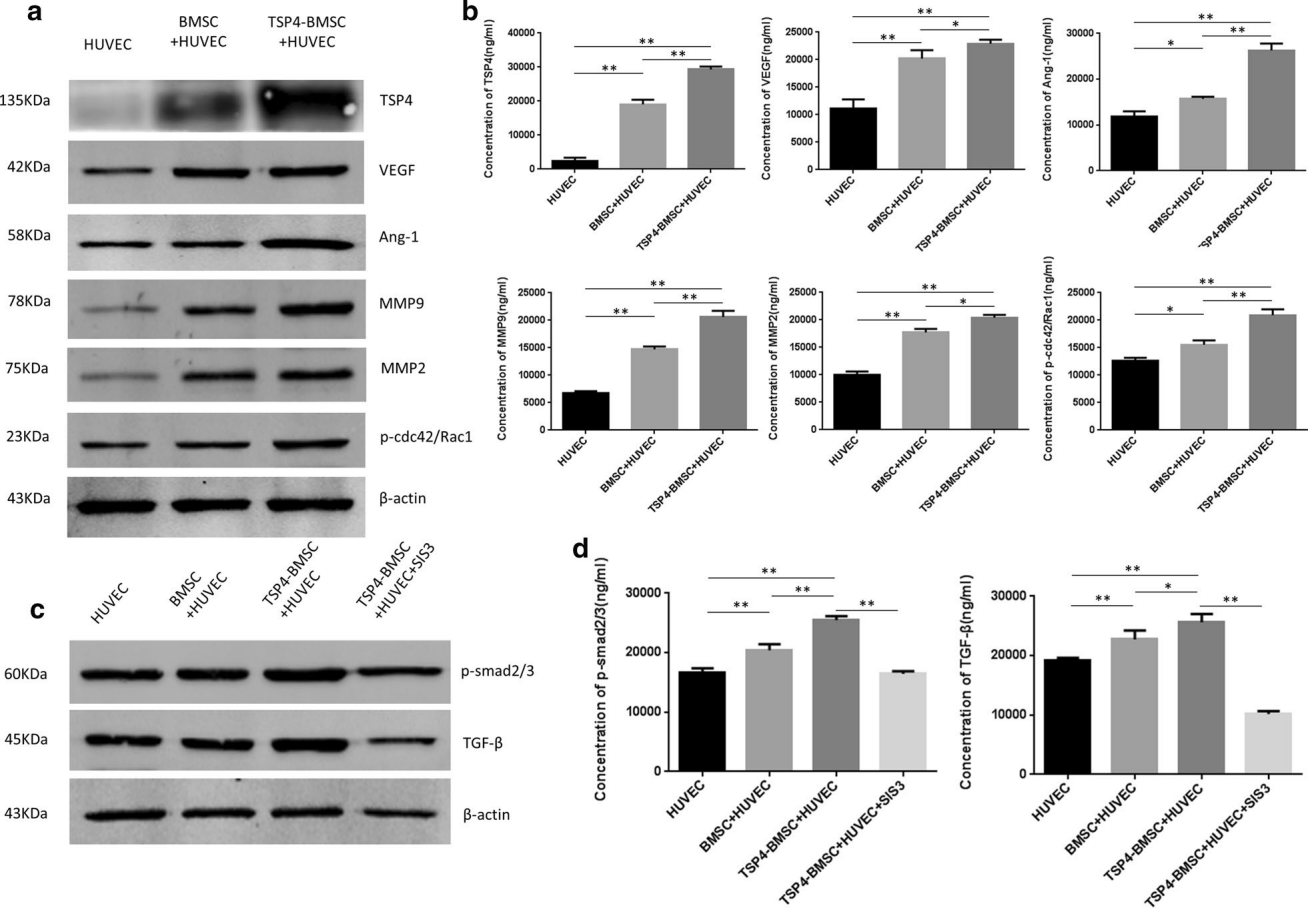
HUVECs incubation with BMSCs induced angiogenic factor expression and activated the TGFβ/Smad2/3 signalling pathway. **a** Western blot assay to determine the expression of HUVEC-derived TSP4, VEGF, Ang-1, MMP9, MMP2 and p-cdc42/Rac1 after incubation with BMSCs for the three groups (HUVEC, BMSC + HUVEC and TSP4-BMSC + HUVEC). **b** Representative western blot results of TSP4, VEGF, Ang-1, MMP9, MMP2 and p-cdc42/Rac1 and quantitative analysis at 48 h after co-incubation. Data are expressed as the mean ± SD. **p < 0.01, *p < 0.05. **c** Representative western blot results for p-Smad2/3 and TGFβ in HUVECs incubated with BMSCs in the four groups (HUVEC, BMSC + HUVEC, TSP4-BMSC + HUVEC and TSP4-BMSC + HUVEC + SIS3); **d** quantitative analysis is presented. Data are expressed as the mean ± SD. **p < 0.01, *p < 0.05

In the current study, treatment with TSP4-BMSCs increased the expression of TSP4 in HUVECs compared to the control and BMSC groups at 48 h (**p < 0.01). We further examined the protein expression of angiogenic factors VEGF, Ang-1, MMP9, and MMP2 in HUVECs after incubation with CM derived from TSP4-BMSCs or BMSCs alone. These angiogenic factors in HUVECs were significantly enhanced in the presence of CM (**p <0.01, *p < 0.05). As shown in Fig. 3a, b, quantitative analysis indicated that the expression of phosphorylated Cdc42/Rac1 in the TSP4-BMSC group was significantly higher than that of the control and BMSC groups (**p < 0.01, *p < 0.05). The increased expression of these angiogenic factors results from the promotion of endothelial cell angiogenesis by incubation with TSP4-BMSCs.

### TSP4-BMSCs activated the TGF-β/Smad2/3 signalling pathway in HUVECs

To further study the intracellular mechanism of TSP4-BMSC’s role in angiogenesis, we investigated the activation of TGF-β/Smad2/3 signalling pathway proteins in HUVECs by western blot assay. As shown in Fig. 3c, d, the expression of phosphorylated Smad2/3 in the TSP4-BMSC group increased significantly after 48 h of treatment (**p < 0.01). Similarly, HUVECs incubated with the CM of the TSP4-BMSC group showed significantly higher expression of TGF-β than that in cells incubated with the CM of BMSCs and control (**p < 0.01). HUVEC angiogenesis in the CM of the TSP4-BMSC group was effectively inhibited in the presence of SIS3 (a Smad2/3 inhibitor) and significantly lower than that of cells in CM of the TSP4-BMSC group (**p < 0.01). This result may be attributed to the counteracting effect of SIS3 on the activation of the Smad2/3 signalling pathway and expression of TGF-β. The above results demonstrated that TSP4-overexpressing BMSCs can obviously activate the TGF-β/Smad2/3 signalling pathway.

### Permanent MCAO model establishment and TSP4-BMSC treatment improves neurological function

**Fig. 4:**
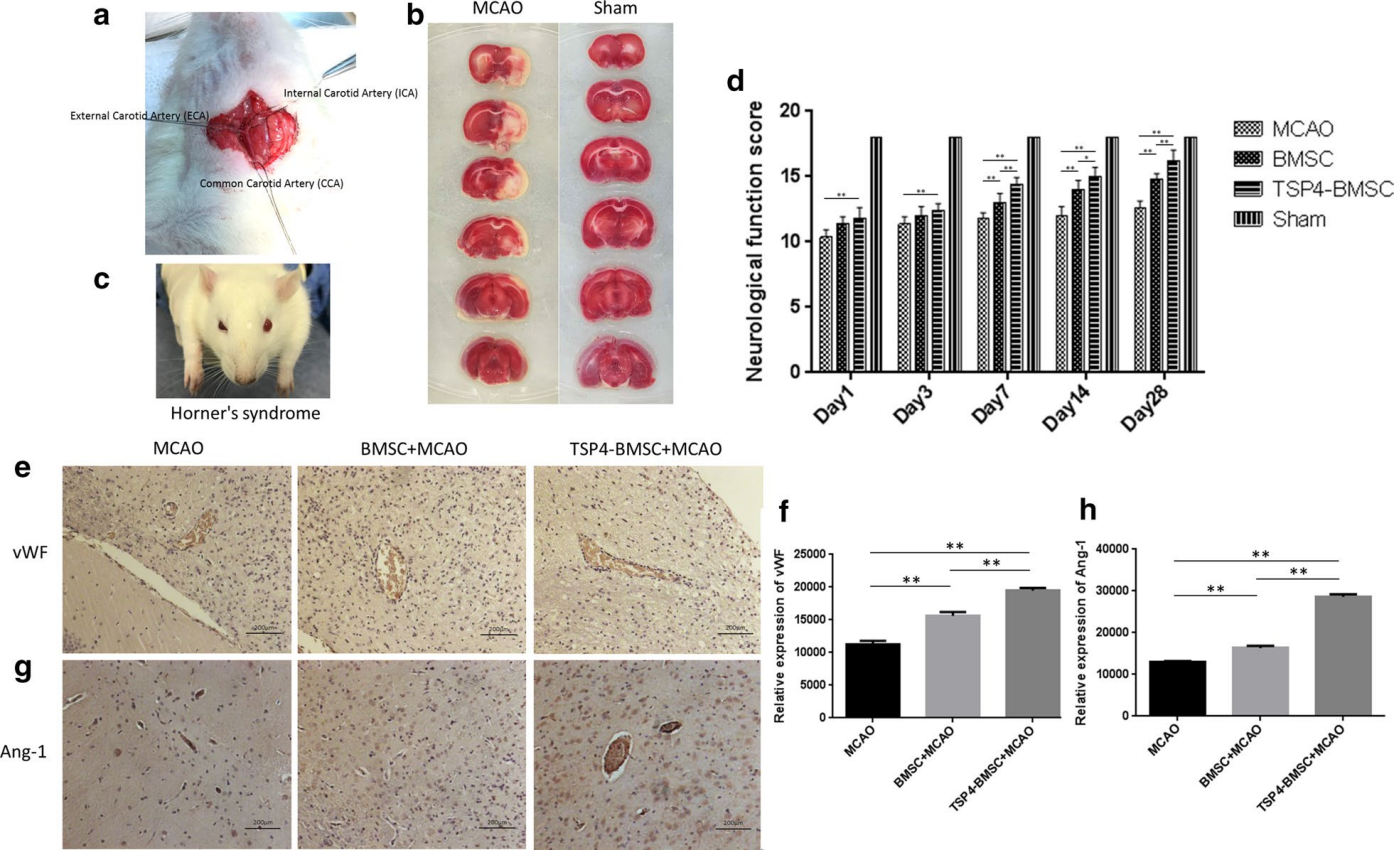
Establishment of a permanent middle cerebral artery occlusion (MCAO) model and expression of vWF and Ang-1 in ischemic brain penumbra of rats. **a** Schematic diagram of the internal carotid artery (ICA), external carotid artery (ECA) and common carotid artery (CCA). **b** Infarction volume on the first day after ischemic stroke. TTC staining of brain slices in the MCAO and sham groups is presented. **c** Schematic diagram of Horner’s syndrome in rat. Horner’s syndrome is characterized by partial ptosis (a weak, droopy eyelid) that occurs on the same side (ipsilateral) as the lesion of the sympathetic trunk in rat. **d**–**g** Immunohistochemistry staining of vWF and Ang-1 is shown, and positive signals were analysed on day 28. Data are expressed as the mean ± SD. **p < 0.01. The experiment was repeated 3 times, and representative pictures are shown. Scale bar, 200 μm

In the present study, we successfully established a permanent MCAO model. Our drop-out rate was 8% due to the exclusion of unstandardized experimental animals, while our animal survival rate was approximately 85%. After operation, the contralateral limb movement of rats was disadvantageous, sensation on the affected side was decreased, and Horner’s syndrome appeared on the face (Fig. 4c). Horner’s syndrome is a combination of symptoms that arises when a group of nerves known as the sympathetic trunk is damaged. TTC staining was performed on rat cerebral slices after operation. A decrease of dehydrogenase activity in ischemic tissues on the operation side were white (Fig. 4b), indicating that the model was successfully produced.

The Garcia JH test is graded on a scale of 0–18, with a higher score indicating minor sensory motor deficits. The TSP4-BMSC group had a significantly improved functional outcome compared with the MCAO group from 1 to 28 days (**p < 0.01). The neurological functional scores in the TSP4-BMSC group continued to increase from 7 to 28 days compared with the BMSC group after operation (**p < 0.01, *p < 0.05), and the BMSC showed a better response to neurological injury than the MCAO group from 7 to 28 days (**p <0.01). The results illustrated that TSP4-BMSCs can improve neurological functional recovery compared with BMSCs and the MCAO model after ischemic stroke.

### TSP4-BMSCs increased angiogenesis in the IBZ

Angiogenesis is defined as the sprouting of new vessels from pre-existing vessels and results in new capillary networks. Our immunohistochemistry results indicated that the treatment of BMSCs alone significantly increased the density of vWF^+^ endothelial cells in the IBZ compared with the MCAO group (**p < 0.01). Moreover, the vWF^+^ cell density was significantly higher in rats treated with TSP4-BMSCs than in those treated with BMSCs alone (**p < 0.01) (Fig. 4e, f). Ang-1 plays a primary role in facilitating angiogenesis. In Fig. 4g, h, the expression of Ang-1 in TSP4-BMSCs was higher than that in the BMSC group (**p < 0.01), and both the TSP4-BMSC and BMSC groups had dramatically improved Ang-1 expression compared with the MCAO group (**p < 0.01). These results illustrated that TSP4-BMSC treatment significantly increased angiogenesis post-stroke.

### TSP4-BMSCs enhanced MMP2 and MMP9 expression in the IBZ

**Fig. 5:**
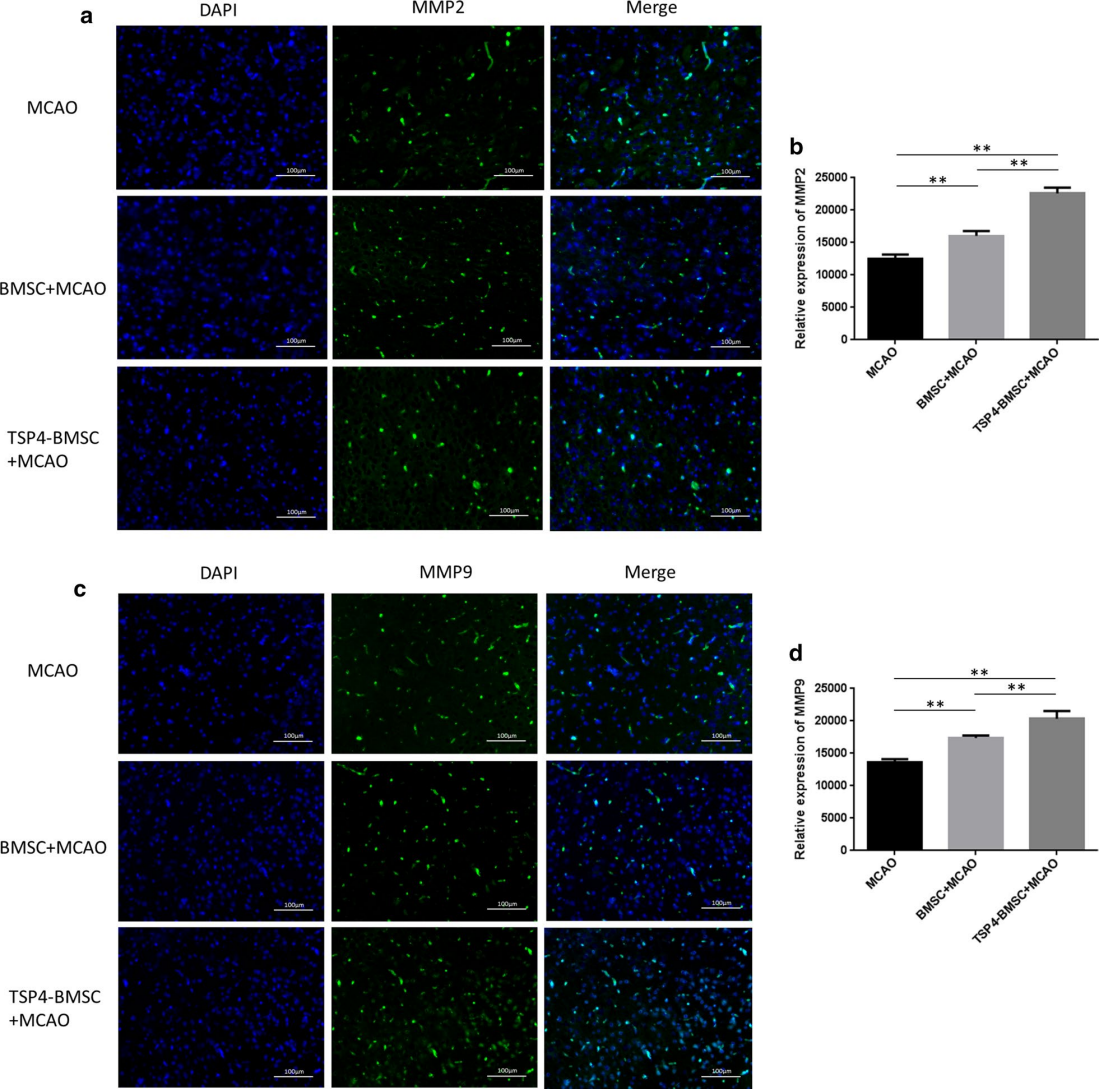
TSP4-BMSCs enhanced angiogenesis by promoting MMP2 and MMP9 expression in the ischemic brain penumbra of rats. **a**, **b** Immunofluorescence staining of MMP2 (green) (scale bar 100 μm) is shown, and the relative expression of VEGF was quantified. Data are expressed as the mean ± SD. **p < 0.01. **c**, **d** Immunofluorescence staining of MMP9 (green) (scale bar, 100 μm) is shown, and relative expression of MMP9 was quantified. Data are expressed as the mean ± SD. **p < 0.01. The experiment was repeated 3 times, and representative pictures are shown. Phenotype identification of BMSCs and TSP4-BMSCs. **a** BMSCs from passage 3 were incubated with fluorescence-conjugated antibodies, including CD90-PerCP, CD45-Alexa, CD34-PE and PBS. The positive expression of CD90 was 99.76%, and the negative identification of CD45 and CD34 was 2.74 and 3.55%, respectively. **b** TSP4-BMSCs were incubated with fluorescence-conjugated antibodies, including CD90-PerCP, CD45-Alexa, CD34-PE and PBS. The positive expression of CD90 was 99.56%, and the negative identification of CD45 and CD34 was 4.97 and 5.97%, respectively

MMP2 and MMP9 are thought to be important factors in angiogenesis because they preferentially degrade basement membrane components, such as type IV collagen [20]. As shown in Fig. 5, the expression of MMP2 and MMP9 (green) was detected by immunofluorescence assay in the IBZ of the brain on day 28 after stroke. A quantitative analysis showed that MMP2 and MMP9 expression in the BMSC group increased markedly compared with that in the MCAO group (**p < 0.01). Furthermore, TSP4-BMSCs dramatically enhanced the expression of MMP2 and MMP9 compared to the BMSC group (**p < 0.01). The results verified that TSP4-BMSC treatment significantly increased the secretion of MMP2 and MMP9 post-stroke.

## Discussion

The present evidence indicates that angiogenesis is very important for the recovery of neurological function post-stroke because angiogenesis results in the formation of new blood vessels, which increases oxygen, glucose, nutrients and neural stem cells (NSCs) migration to the IBZ [21]. BMSCs are a candidate for ischemic stroke treatment in terms of their multi-potentiality [11]. Several studies have shown that BMSCs can significantly enhance endogenous angiogenesis, as well as express neuronal and endothelial phenotypes in the IBZ after stroke [12, 14]. However, many problems remain unresolved. In practical applications, the survival rate of BMSCs after transplantation and the rate of angiogenesis is reduced due to changes in the microenvironment, such as oxidative stress and hypoxia, which greatly limits the therapeutic effect of stem cell transplantation. Therefore, improving the efficiency of angiogenesis in BMSC treatment is key for transplantation of stem cells after ischemic stroke. Data show that modification of BMSCs with neuroprotective factor or neurotrophic factor coding gene can improve the therapeutic effect [22, 23]. Thrombospondins are a family of extracellular, multidomain, oligomeric and calcium-binding glycoproteins that regulate various cell interactions [24]. Thrombospondins are conserved proteins with regions of high sequence identity but distinct temporal expression, cellular distribution and functional capabilities, which are enabled by interaction with a large number of proteins and proteoglycans. Recent studies have observed the presence and critical roles of TSP4 in the heart, blood vessels, and vascularized tissues [25, 26], which are related to angiogenesis.

Our results showed that TSP4-overexpressing BMSCs could maintain the stem cell phenotype and produce TSP4 target protein both intracellularly and extracellularly. In co-culture with HUVECs, the expression of TSP4 increased not only in HUVECs but also in the co-culture supernatant. The mechanisms underlying vascular diseases have mostly focused on the cells involved. Previous work has shown that TSP4 may contribute to ECM structure and function [27]. The ECM is clearly an important regulator of vascular pathologies, but it has only recently become appreciated as a target for pharmacotherapy [28]. Therefore, *tsp4* gene-modified BMSCs not only promote the expression of TSP4 in endothelial cells but more importantly in the ECM.

Angiogenesis is critical for recovering neurological functional post-stroke [29]. Blood vessel formation allows blood flow in the ischemic penumbra, which may protect the ischemic brain from injury. Angiogenesis is a process by which new blood vessels are formed from pre-existing vascular structures, which leads to the reestablishment of the blood supply to the brain after ischemia [30]. Increased angiogenesis is an effective method to improve the prognosis of patients with stroke [31]. At present, angiogenesis in vitro may be expressed as a tubular structure of endothelial cells, and the total length of the structure may be evaluated [32]. To further observe the effect of TSP4-BMSCs on the angiogenesis of endothelial cells, we performed a series of wound healing, tube formation, and arterial ring experiments, and the results showed that TSP4-BMSCs could significantly promote the migration, proliferation and angiogenesis of endothelial cells and tube formation compared with only BMSC treatment. Both VEGF and TGF-β are central to the processes of angiogenesis, tissue inflammation, and fibrosis. VEGF is a pleiotropic angiogenic growth factor that stimulates the proliferation of vascular endothelial cells [33]. In addition, VEGF plays critical roles in neovascular remodelling in ischemic stroke [34]. TGF-β may play a modulatory role in angiogenesis, inflammation, and tissue remodelling after ischemic insult [35]. The ELISA results showed that in addition to an increase in TSP4 expression, the expression of VEGF and TGF-β was significantly increased in the supernatant of co-cultured endothelial cells and TSP4-BMSCs.

Though the defined mechanism of BMSC treatment for stroke remains ambiguous, most experimental results have suggested that the therapeutic effects probably relate to the paracrine function of BMSCs, allowing the secretion of different types of trophic factors and resulting in synaptogenesis, neurogenesis and angiogenesis [36, 37]. Previous evidence indicated that intravenously injected BMSCs are located in peripheral organs (lungs, spleen, and liver) rather than the brain of the MCAO model [38]; thus, the amelioration of neurological function by BMSCs is related to their paracrine function instead of their engraftment into the infarction zone. Simultaneously, our western blot results showed that TSP4-BMSCs could promote the expression of angiogenic factors, such as VEGF, Ang-1, MMP9, and MMP2, in endothelial cells. Previous data showed that the VEGF/VEGFR2 signalling pathway and EPC contributed to angiogenesis after injury [39]. Ang-1 binds to its specific receptor Tie-2 and recruits mural cells to wrap around endothelial cells, thereby ensuring the eventual maturation and stabilization of new blood vessels [40]. MMP-9 can promote the release of VEGF [41], which might enhance the proliferation of vascular epithelial cells and inhibit their apoptosis after injury [42]. MMP2, a member of the MMP family, can recognize various extracellular matrix components as substrates [43] to enhance aberrant tumour angiogenesis and metastasis [44]. Current preclinical evidence further highlighted that decreasing MMP-2 activity by specific inhibitors led to attenuated angiogenesis and tumour progression [45]. The above results further explain that TSP4-BMSCs may promote the expression of angiogenic factors in endothelial cells and the extracellular space. Therefore, the TSP4 gene modification effect may be due to promoting the paracrine effect of BMSCs. In addition, the application of lentiviral infection cell therapy has proven to be safe in human experiments, so this method has certain practical significance for the treatment of patients with ischemic stroke [46]. It has been reported that the optimal dose of BMSC transplantation for ischemic stroke is from 1 × 10^6^ to 10^7^ [47]. Based on previous experience [48], we have chosen TSP4-BMSC transplantation dose of 2 × 10^6^.

In summary, the application of BMSCs as a TSP4 gene therapy platform can not only improve the sustained secretion of TSP4, achieve local stable therapeutic concentration, enhance the effect of local angiogenesis but also ameliorate the microenvironment after ischemia by promoting angiogenesis and providing the optimal space for the proliferation and differentiation of BMSCs. TSP4 can also magnify the paracrine effect of BMSCs, reduce the apoptosis of BMSCs, improve the homing and survival rate of BMSCs after transplantation, and effectively exert a synergistic effect of TSP4 and BMSCs in angiogenesis. However, crucial problems still need to been solved, such as the engraft time window, the quantity of TSP4-BMSCs to transplant and transplantation approaches. Our experimental evidence indicated that TSP4 is an effective promoter of angiogenesis in BMSCs. However, whether TSP4 can be expressed stably in regenerating BMSCs is unclear. Some possible side effects must be considered when this strategy is applied. A series of clinical trials on BMSC in the treatment of ischemic stroke have been carried out, which proves the safety and efficacy of cellular therapy. However, patients should also pay close attention to the recovery of the nervous system and other basic signs to avoid adverse events [49–51]. Because accelerating plaque angiogenesis can lead to the expansion and rupture of a plaque, the potential risk of an angiogenesis-borne disease exists and includes tumour formation and metastasis, the aggravation of diabetic retinopathy, etc. Thus, we will investigate the combination of BMSCs and TSP4 in angiogenesis and homeostasis in future research.

## Conclusion

In summary, our present study firstly demonstrates that TSP4-overexpressing BMSCs can improve angiogenesis in IBZ and improves neurological function post-stroke. The therapeutic effects are associated with the paracrine function of TSP4-BMSC and activation of TGF-β/Smad2/3 signalling pathway.

## Additional files

**Additional file 1.** Phenotype identification of BMSCs and TSP4-BMSCs. (**a**) BMSCs from passage 3 were incubated with fluorescence-conjugated antibodies, including CD90-PerCP, CD45-Alexa, CD34-PE and PBS. The positive expression of CD90 was 99.76%, and the negative identification of CD45 and CD34 was 2.74 and 3.55%, respectively. (**b**) TSP4-BMSCs were incubated with fluorescence-conjugated antibodies, including CD90-PerCP, CD45-Alexa, CD34-PE and PBS. The positive expression of CD90 was 99.56%, and the negative identification of CD45 and CD34 was 4.97 and 5.97%, respectively.

**Additional file 2.**  One video clip of the focal cerebral ischemia rat model is presented to confirm the establishment of disease model.

**Additional file 3.**  One video clip of the control rat model is presented to compare the behaviour between the disease rat model and normal rat.

## Abbreviations

BMSCs: bone marrow stromal cells
TSP4: thrombospondin-4
HUVECs: human umbilical vein endothelial cells
VEGF: vascular endothelial growth factor
TGF-β: transforming growth factor-β
Ang-1: angiopoietin-1
MMP9: matrix metalloprotein 9
MMP2: matrix metalloprotein 2
vWF: von Willebrand factor
ELISA: enzyme-linked immunosorbent assay
SD: Sprague–Dawley
PBS: phosphate buffer saline
DMEM: Dulbecco’s modified Eagle’s medium
FBS: foetal bovine serum
MCAO: middle cerebral artery occlusion
IBZ: ischemic boundary zone
CM: conditioned media
NSCs: neural stem cells
