# Supplementary figures and images for: Promoting therapeutic angiogenesis of focal cerebral ischemia using thrombospondin-4 (TSP4) gene-modified bone marrow stromal cells (BMSCs) in a rat model
Source: J Transl Med. 2019 Apr 4;17:111. doi: 10.1186/s12967-019-1845-z (PMC6449913; doi:10.1186/s12967-019-1845-z)

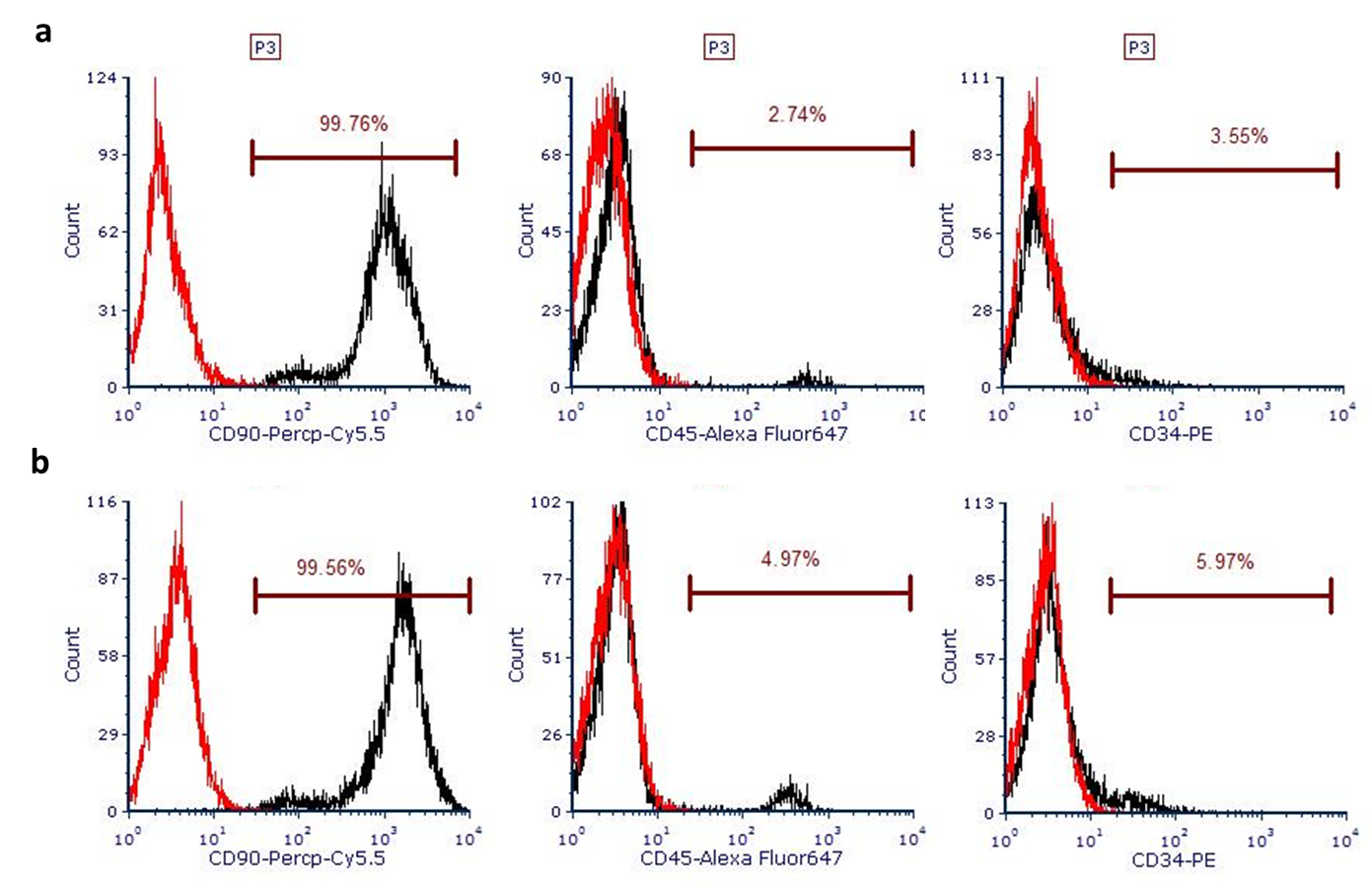

Supplement: Supplementary file 1 — Additional file 1. Phenotype identification of BMSCs and TSP4-BMSCs. (a) BMSCs from passage 3 were incubated with fluorescence-conjugated antibodies, including CD90-PerCP, CD45-Alexa, CD34-PE and PBS. The positive expression of CD90 was 99.76%, and the negative identification of CD45 and CD34 was 2.74 and 3.55%, respectively. (b) TSP4-BMSCs were incubated with fluorescence-conjugated antibodies, including CD90-PerCP, CD45-Alexa, CD34-PE and PBS. The positive expression of CD90 was 99.56%, and the negative identification of CD45 and CD34 was 4.97 and 5.97%, respectively. [file 12967_2019_1845_MOESM1_ESM.tif]
